# Ferroptosis regulation by Cap’n’collar family transcription factors

**DOI:** 10.1016/j.jbc.2024.107583

**Published:** 2024-07-16

**Authors:** Magdalena B. Murray, Scott J. Dixon

**Affiliations:** Department of Biology, Stanford University, Stanford, California, USA

**Keywords:** oxidative stress, iron metabolism, lipid peroxidation, nuclear factor 2 (erythroid-derived 2-like factor) (NFE2L2), NRF2, BACH1, NFE2L1, glycosylation

## Abstract

Ferroptosis is an iron-dependent cell death mechanism that may be important to prevent tumor formation and useful as a target for new cancer therapies. Transcriptional networks play a crucial role in shaping ferroptosis sensitivity by regulating the expression of transporters, metabolic enzymes, and other proteins. The Cap’n’collar (CNC) protein NFE2 like bZIP transcription factor 2 (NFE2L2, also known as NRF2) is a key regulator of ferroptosis in many cells and contexts. Emerging evidence indicates that the related CNC family members, BTB domain and CNC homolog 1 (BACH1) and NFE2 like bZIP transcription factor 1 (NFE2L1), also have roles in ferroptosis regulation. Here, we comprehensively review the role of CNC transcription factors in governing cellular sensitivity to ferroptosis. We describe how CNC family members regulate ferroptosis sensitivity through modulation of iron, lipid, and redox metabolism. We also use examples of ferroptosis regulation by CNC proteins to illustrate the flexible and highly context-dependent nature of the ferroptosis mechanism in different cells and conditions.

## Transcription factors and cell death

Cell death is a terminal phenotype important for mammalian development and homeostasis (1). Excessive cell death contributes to neurodegeneration and other pathologies, whereas insufficient cell death may contribute to tumorigenesis. It is therefore important to understand how cell death is regulated in mammalian cells. In addition to apoptosis, cell death can be executed by several nonapoptotic cell death mechanisms, including pyroptosis, necroptosis, and ferroptosis (2). These cell death mechanisms are intricately regulated by posttranslational and metabolic mechanisms (3, 4, 5, 6). Transcriptional networks also play an important role in regulating cell death and are the general focus of this review.

Transcription factors can regulate cell death in different ways. In some settings, transcription factors can induce the expression of prodeath gene products that can act directly to kill the cell. For example, in *Caenorhabditis elegans* germ cells, transcriptional upregulation of *egl-1* and *ced-3* can directly induce apoptosis (7). In mammalian cells, endoplasmic reticulum (ER) stress can result in higher expression of the transcription factor C/EBP homologous protein (CHOP), which in turn activates apoptosis by increasing the expression of death receptor 5 (*DR5*) (8). More subtly, transcription factors can alter the sensitivity of a cell to a distinct lethal stimulus, such as a small molecule. In this paradigm, transcription factors modulate cell death sensitivity positively or negatively by altering the expression of gene products that regulate the cell death mechanism (9). This is how Cap’n’collar (CNC) family transcription factors appear to regulate ferroptosis, a concept we will examine in detail below.

## Ferroptosis from a transcriptional perspective

**Figure 1:**
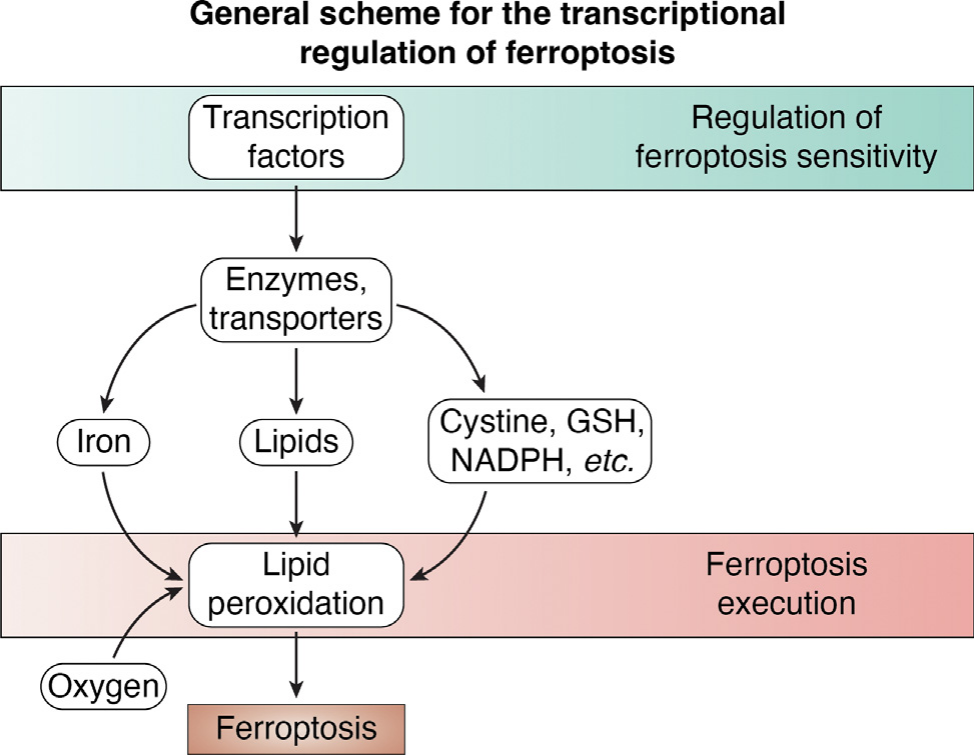
**Overview of the transcriptional regulation of cell death.** Overview of the relationship between transcription factor-mediated regulation of ferroptosis sensitivity and the execution of ferroptosis due to the oxygen- and iron-dependent accumulation of lipid peroxides.

Ferroptosis is an iron-dependent form of nonapoptotic cell death (5, 10). Metabolic pathways involved in iron, lipid, and redox metabolism govern the execution and regulation of ferroptosis (Fig. 1). The execution of ferroptosis involves iron-dependent peroxidation of polyunsaturated fatty acid (PUFA)-containing phospholipids (11, 12). Rising levels of plasma membrane phospholipid peroxidation result in membrane stiffening, Na^+^ and Ca^2+^ influx, K^+^ efflux, and membrane rupture (13). This process is normally restrained by enzymes that can stop the propagation of lipid peroxidation reactions. Glutathione peroxidase 4 (GPX4) appears to be the most critical negative regulator of ferroptosis in most cells, and the target of important ferroptosis-inducing small molecules (14, 15, 16). GPX4 uses reduced glutathione (GSH) to convert potentially toxic lipid hydroperoxides (L-OOH) to nontoxic lipid alcohols (L-OH). Ferroptosis suppressor protein 1 (FSP1, encoded by the gene *AIFM2*) is another suppressor of lipid peroxidation. FSP1 is an NAD(P)H-dependent enzyme that (re)generates reduced endogenous lipophilic radical trapping antioxidants, including coenzyme Q10 (CoQ10) and vitamin K (17, 18, 19, 20), which suppress lipid peroxidation. Several additional enzymes, including GTP cyclohydrolase 1 (GCH1) and dihydroorotate dehydrogenase (DHODH), and metabolites, including 7-dehydrocholesterol (7-DHC), can also suppress ferroptosis by directly or indirectly inhibiting lipid peroxidation (21, 22, 23).

PUFA-containing phospholipids are oxidized during the execution of ferroptosis—a process that drives the terminal permeabilization of the plasma membrane (12, 13, 21, 24). Enzymes required for the synthesis of PUFA-containing phospholipids, such as acyl-CoA synthetase long chain family member 4 (ACSL4), are therefore often essential for ferroptosis (11, 25, 26). In contrast to more oxidizable PUFA-containing phospholipids, less oxidizable monounsaturated fatty acid (MUFA)-containing phospholipids, where the MUFAs are taken up from the environment or synthesized by stearoyl-CoA desaturase (SCD), prevent ferroptosis by limiting lipid peroxidation (14, 27, 28, 29). Membrane-bound O-acyltransferase domain-containing 1 (MBOAT1) and MBOAT2 suppress ferroptosis sensitivity through the incorporation of MUFA-CoA into anti-ferroptotic MUFA-containing phospholipids (14, 29). Depending on the cell type and context, different metabolites and proteins assume greater importance in ferroptosis regulation (20, 26, 30, 31). Overall, hundreds of metabolites and proteins likely regulate ferroptosis sensitivity to some degree in every cell (32).

There is currently little evidence that transcription factor-mediated gene induction or gene repression alone is sufficient to induce ferroptosis. There is, however, abundant evidence that transcription factors can alter ferroptosis sensitivity by regulating the levels of proteins, including metabolite transporters and enzymes, that in turn alter the sensitivity of the cell to ferroptosis induction by other stimuli (*e.g.*, a small molecule inhibitor of system x_c_^-^ or GPX4) (32, 33, 34, 35, 36, 37). Below, we specifically focus on the function of CNC family transcription factors and their role in ferroptosis regulation. Collectively, the goal of this review is to describe how CNC transcription factors regulate ferroptosis and to illuminate broader themes about the transcriptional regulation of this cell death process.

It is useful to examine the transcriptional regulation of ferroptosis by CNC proteins for several reasons. First, three members of the CNC family now have defined roles in ferroptosis regulation; it is useful to summarize recent findings. Second, exploring known or postulated mechanisms of ferroptosis regulation by CNC proteins helps to understand how ferroptosis sensitivity is regulated by transcriptional processes. Third, individual CNC transcription factors appear to have variable effects on ferroptosis, which sometimes depend on the cell type and/or condition, highlighting the context-dependent nature of this cell death mechanism.

## Transcriptional regulation by the Cap’n’collar family

**Figure 2:**
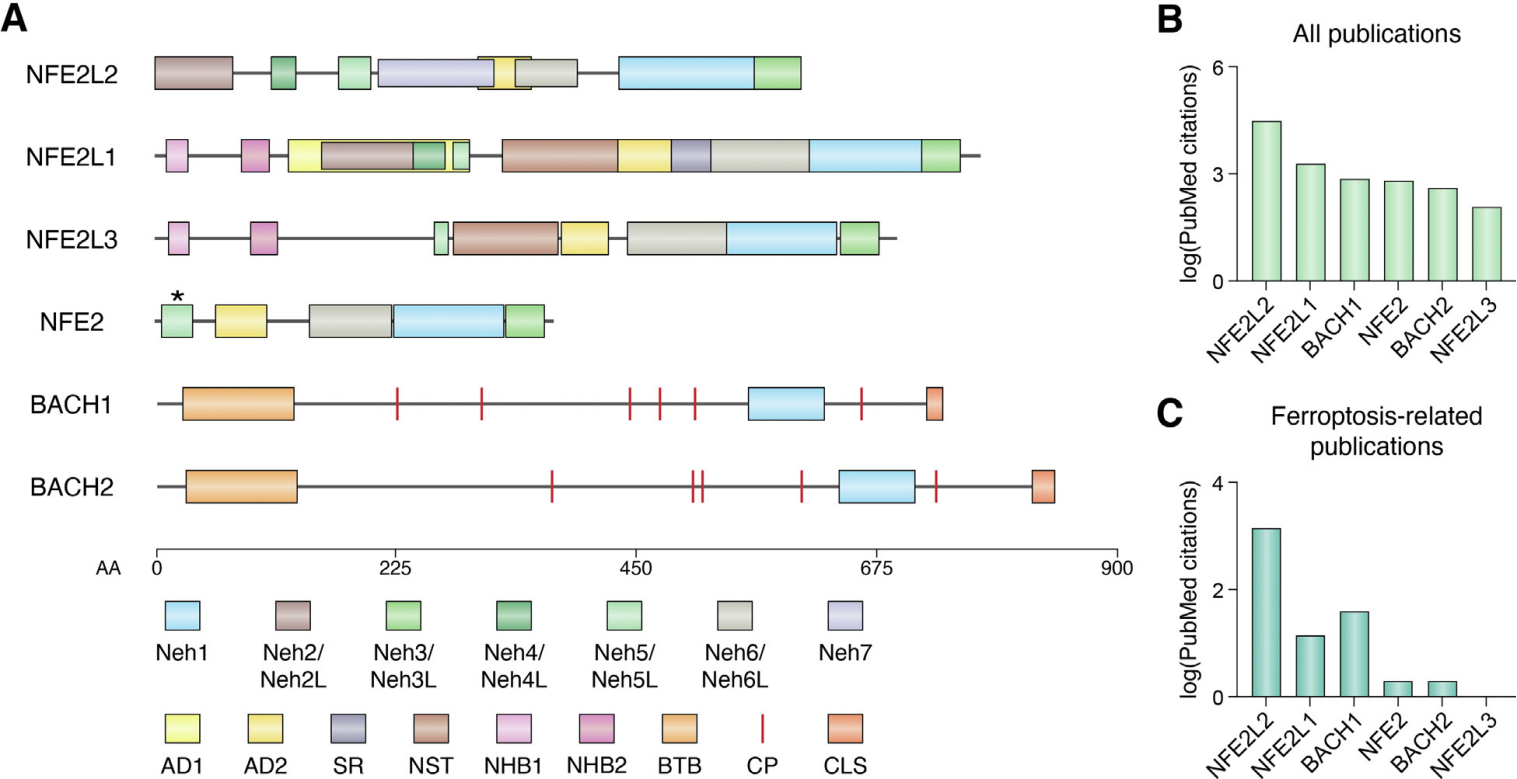
**Comparison of CNC family member domain structures and PubMed citations.***A*, depiction of the domain architecture of mammalian CNC proteins. *Asterisk* indicates that the position of the given domain has not been published. The common CNC and DNA binding domains are located within the Neh1 domain, depicted in *light blue*. ‘L’ denotes ‘-like’ *B*, the log_10_-transformed number of publications listed in PubMed in which the abbreviated name of a given CNC protein is included in the publication title and/or abstract. *C*, the number of publications listed in PubMed (log_10_-transformed) in which the name of a given CNC protein as well as the term “ferroptosis”, are included in the publication title and/or abstract. Note that for NFE2 in panels *B* and *C*, only publications whose title and/or abstract did not refer to NFE2L1, NFE2L2, and NFE2L3 were counted, as “NFE2” is entirely embedded within the unabbreviated names for these proteins. CNC, Cap'n’collar; NFE2, nuclear factor, erythroid E; CP, Cys-Pro motif; CLS, cytoplasmic localization sequence.

In mammals, the CNC family of transcription factors comprises six proteins: NFE2 like bZIP transcription factor 1 (NFE2L1), NFE2L2, NFE2 like bZIP transcription factor 3 (NFE2L3), nuclear factor, erythroid 2 (NFE2), BACH1, and broad-complex, tramtrack and bric-a-brac (BTB) domain and CNC homolog 2 (BACH2) (Fig. 2*A*). CNC orthologs are also found in simpler metazoans, including *C. elegans* and *Drosophila melanogaster* (38), but in this review, we will focus on the mammalian proteins. All six proteins share a common CNC sequence adjacent to a DNA binding domain. Note that there can be some confusion with gene and protein nomenclature in this field. The transcription factor nuclear respiratory factor 1 (NRF1) is distinct from the CNC transcription factor NFE2L1 (often abbreviated NRF1). For this reason, in this review we endeavor to use standardized gene and protein names (*e.g.*, NFE2L1 and NFE2L2) wherever possible to avoid confusion.

**Figure 3:**
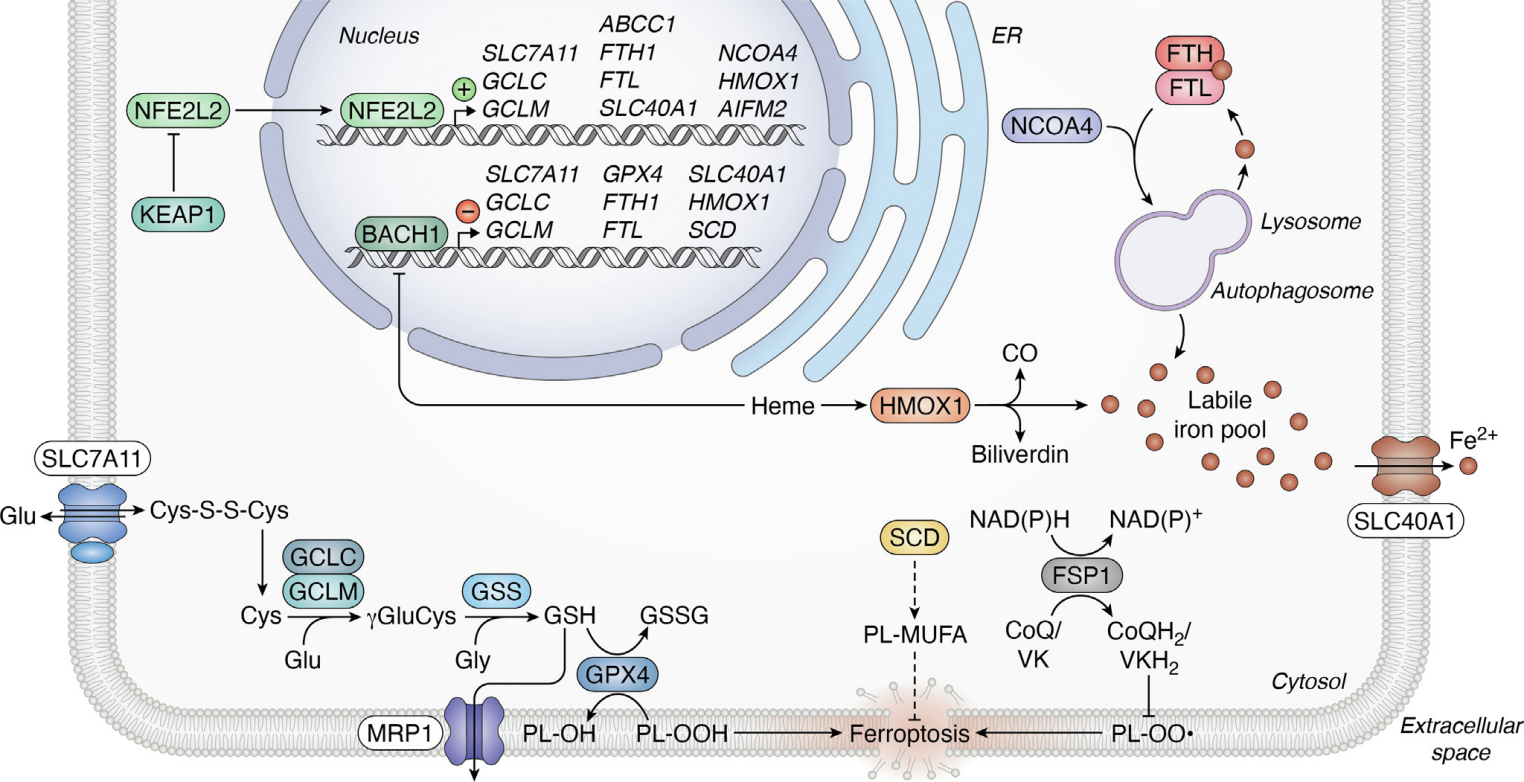
**Regulation of ferroptosis by NFE2L2 and BACH1.** NFE2L2 and BACH1 are negatively regulated at the posttranslational level by KEAP1 and heme, respectively. However, in conditions that promote the stability of these transcription factors, NFE2L2 and BACH1 can directly upregulate and downregulate, respectively, the expression of several genes with known roles in several ferroptosis regulatory pathways. The glutamate/cystine antiporter x_c_^-^ imports cystine into the cell through the SLC7A11 subunit. Cystine can be reduced and subsequently incorporated into the antioxidant glutathione (GSH) by the enzyme glutamate-cysteine ligase, comprised of the subunits GCLC and GCLM, as well as by GSS. GSH can be used as a cosubstrate by the enzyme GPX4 to reduce proferroptotic lipid hydroperoxides to lipid alcohols, thereby inhibiting ferroptosis. GSH can be exported from the cell by MRP1, encoded by *ABCC1*. FSP1, encoded by *AIFM2*, acts as a radical trapping agent by (re)generating reduced CoQ10 or reduced vitamin K. Regulation of lipids themselves can also modulate ferroptosis sensitivity. SCD synthesizes MUFAs that can be incorporated into antiferroptotic phospholipids in the plasma membrane. Finally, iron is involved in the generation of lipid peroxides. The labile iron pool is impacted by several pathways. Iron can be exported by SLC40A1, generated by the HMOX1-mediated breakdown of heme, or generated by the autophagosome-mediated degradation of ferritin (comprised of FTH1 and FTL). It is through these pathways that NFE2L2 and BACH1 likely regulate ferroptosis. BACH1, BTB domain and CNC homolog 1; FSP1, ferroptosis suppressor protein 1; FTL, ferritin light chain; HMOX1, heme oxygenase 1; KEAP1, kelch like ECH associated protein 1; MRP1, multidrug resistance-associated protein 1; MUFA, monounsaturated fatty acid; NFE2L2, NFE2 like bZIP transcription factor 2; SCD, stearoyl-CoA desaturase; VK, vitamin K.

**Figure 4:**
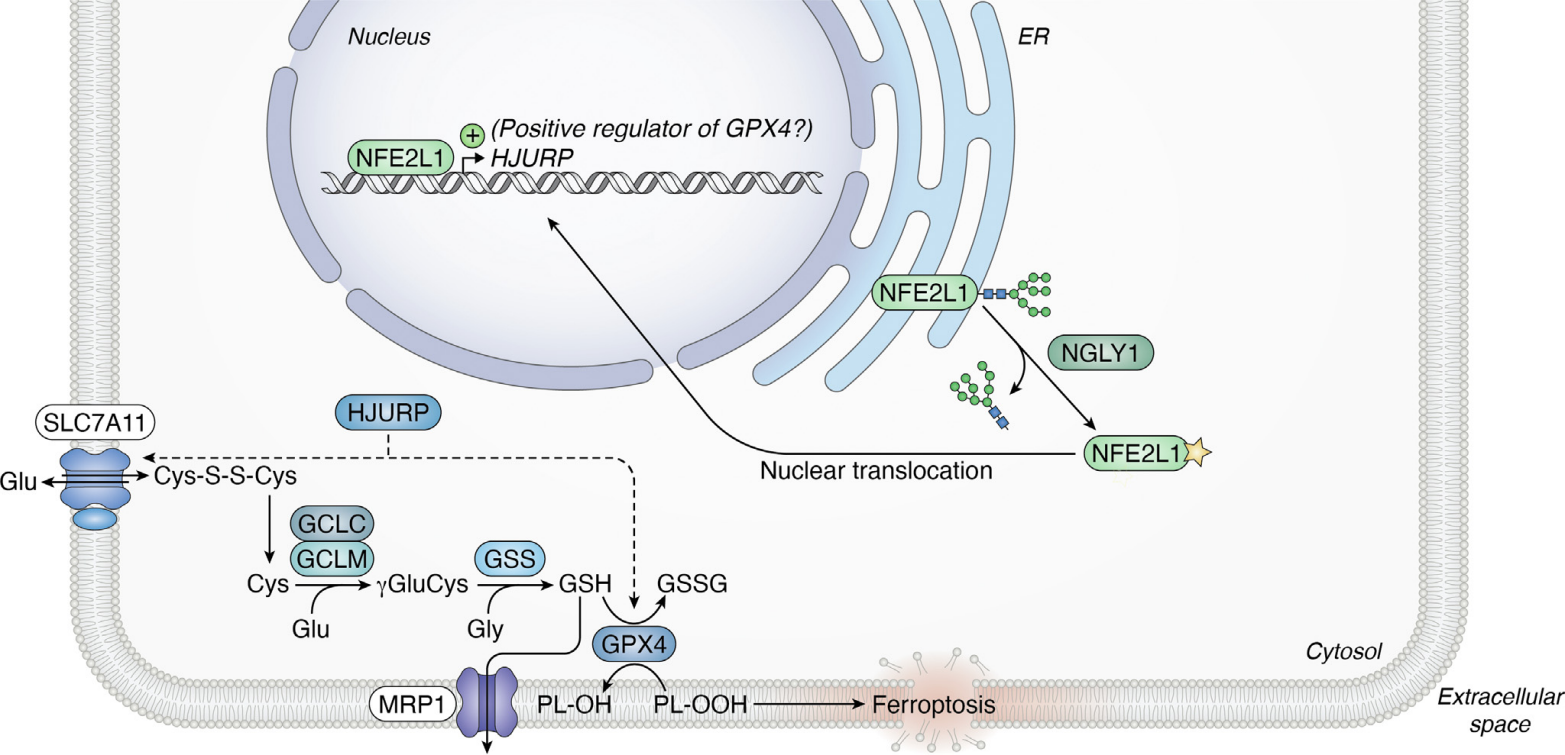
**Regulation of ferroptosis by NFE2L1.** While NFE2L1 can bind several ferroptosis-related genes, only *HJURP* has been identified as a direct transcriptional target of NFE2L1 in the context of ferroptosis. HJURP, through a mechanism that has yet to be defined, likely contributes to NFE2L1-mediated SLC7A11 and GPX4 upregulation. NFE2L1 indirectly regulates GPX4, presumably by upregulating the expression of a gene that increases GPX4 protein synthesis or stability. NGLY1, an enzyme that catalyzes deglycosylation of NFE2L1 and promotes NFE2L1 nuclear localization, can promote ferroptosis resistance in an NFE2L1-dependent manner in some cells. The *yellow star* indicates that aspartic acid is generated by NGLY1-mediated deglycosylation at a former asparagine residue. HJURP, Holliday junction recognition protein; NFE2L1, NFE2 like bZIP transcription factor 1.

The abundance and function of CNC family proteins are regulated *via* distinct mechanisms. Several CNC transcription factors are stress-responsive proteins that are basally translated but then quickly degraded, accumulating to high levels only under conditions where constitutive turnover mechanisms are inhibited by specific stressors. NFE2L2 is degraded by the proteasome in a manner dependent upon the kelch like ECH associated protein 1 (KEAP1) E3 ubiquitin ligase (39, 40) (Fig. 3). NFE2L1 and NFE2L3 are N-glycosylated in the ER, then retrotranslocated to the cytoplasm (41, 42, 43, 44, 45, 46, 47, 48, 49). NFE2L1 is deglycosylated by N-glycanase 1 (NGLY1), a reaction that results in “editing” of formerly N-glycosylated Asn residues to Asp residues (41, 48, 50, 51, 52) (Fig. 4). Both NFE2L1 and NFE2L3 are cleaved from the ER by the protease DNA damage inducible 1 homolog (DDI2) (48, 49, 53, 54). Like NFE2L2, NFE2L1 and NFE2L3 are mostly constitutively degraded by the proteasome under basal conditions (43, 49, 55). All three proteins are stabilized upon proteasome inhibition.

Structurally, CNC proteins are relatively diverse (Fig. 2*A*). NFE2L2 contains seven NRF2-ECH homology (Neh) domains, some of which are shared by other family members. The Neh1 domain harbors the CNC sequence shared by all CNC proteins, as well as a basic-leucine zipper (bZIP) sequence shared by all mammalian members (56, 57). Neh2 or Neh2-like domains, which facilitate KEAP1 binding, are present in NFE2L1 and NFE2L2; KEAP1 binding leads to NFE2L2 ubiquitination and degradation but stabilizes NFE2L1 (57, 58, 59, 60). Neh3/Neh3-like domains are found in all mammalian CNC proteins except BACH1 and BACH2, Neh4/Neh4-like domains are present in NFE2L1 and NFE2L2, and Neh5/Neh5-like domains are shared by all CNC proteins except BACH1 and BACH2 (57, 61). These Neh3, Neh4, and Neh5 domains promote transactivation by binding coactivators (57, 62, 63, 64). Neh6/Neh6-like domains promote beta-transducin repeat-containing E3 ubiquitin protein ligase (BTRC)-mediated degradation and are present in all CNC proteins except BACH1 and BACH2 (57, 65). NFE2L2 uniquely contains an Neh7 domain that promotes binding to the transcription factor retinoic X receptor alpha (RXRA, also known as NR2B1), an interaction that appears to inhibit NFE2L2 activity (57, 66). All CNC members except BACH1 and BACH2 also contain an acidic domain 2 (AD2)/AD2-like domain that promotes target gene upregulation through a mechanism that has yet to be elucidated (56, 57, 67).

Some protein domains are less commonly shared among mammalian CNC members. NFE2L1 uniquely contains a serine-rich domain, which may contribute to transactivation, as well as an AD1 region spanning the Neh2-like, Neh4-like, and Neh5-like domains (56, 57, 67). Among mammalian CNC proteins, the ER membrane-anchoring N-terminal homology box 1 (NHB1) and NHB2 domains are found only in NFE2L1 and NFE2L3 (42, 45, 57, 68). While localized to the ER, NFE2L1 and NFE2L3 are N-glycosylated at their putative or demonstrated asparagine/serine/threonine-rich (NST) domains at NxS/T motifs, where x is any amino acid except proline (45, 46, 47, 57, 69). BACH1 and BACH2 harbor multiple Cys-Pro motifs that bind heme and promote the nuclear export and proteasomal degradation of these proteins (70, 71, 72, 73, 74, 75). BACH1 and BACH2 also contain a BTB domain, which can promote protein-protein binding (76, 77) as well as a C-terminal cytoplasmic localization sequence (78, 79).

To regulate gene expression, CNC proteins bind a common DNA motif termed the antioxidant response element (ARE): 5′-TGABNNNGC-3′, where B indicates C/G/T and N indicates any nucleotide (80). AREs are found in various positions relative to transcription start sites, including proximal and distal promoters, introns, and other genomic regions (81, 82, 83, 84, 85, 86). NFE2L1 and NFE2L3 preferentially bind introns, whereas NFE2L2 shows relatively greater preference for proximal promoters and the first exon of target genes (80). ARE binding specificity is refined by unique preferences among CNC family members for nucleotides adjacent to the ARE (80).

Mammalian CNC transcription factors cannot regulate gene expression as monomers. Canonically, these proteins heterodimerize with small MAF transcription factors, including MAFF, MAFK, and MAFG, to regulate transcription (45, 55, 87, 88, 89, 90, 91, 92). Additional binding partners for some CNC proteins include the AP-1 transcription factor JUN (c-Jun) and the mediator complex subunit 16 (MED16) (facilitated by the CNC Neh4/5 and Neh1 domains) (93, 94, 95). CNC family members also form complexes with epigenetic modifiers. NFE2L1 can bind the chromatin remodeling complex TIP60 to upregulate target gene expression (96). The Neh3 domain of NFE2L2 can bind chromodomain helicase DNA binding 6 (CHD6), an interaction that appears to increase target gene transactivation (62). NFE2L2 can also form a complex with the switch/sucrose nonfermentable (SWI/SNF) complex through the intermediary special AT-rich sequence binding protein 2 (SATB2) to promote target gene expression (97). In another example, the BACH1 BTB domain can bind histone deacetylase 1 (HDAC1) to repress target gene expression (77, 98, 99). To what extent these cofactors can be bound by other CNC proteins is unclear.

Finally, CNC transcription factor stability and activity are modulated by posttranslational modifications (PTMs). As noted above, ubiquitination and subsequent proteolysis is a major negative regulator of certain CNC proteins. By contrast, NFE2L2 acetylation by CREB binding protein (CBP)/p300 can promote NFE2L2 nuclear localization, ARE binding, MAFG cofactor binding, and transcriptional activity (100, 101, 102). NFE2L2 phosphorylation by the serine/threonine kinase AMP-activated protein kinase (AMPK) can also increase NFE2L2 nuclear localization (103). Conversely, phosphorylation of NFE2L2 by the serine-threonine kinase glycogen synthase kinase 3 beta (GSK-3β) can result in NFE2L2 nuclear export and degradation (65, 104). The presence of sugar moieties can repress the activity of a few CNC proteins. For instance, glycation (the nonenzymatic attachment of sugars to a protein) can reduce NFE2L2 stability, transcriptional activity, and binding to MAFG (105). Unlike glycation, N-glycosylation involves the enzymatic attachment of N-glycans to Asn residues. This PTM promotes NFE2L1 retention in the ER, thereby inhibiting the nuclear localization of NFE2L1 in response to proteotoxic stress (41). N-glycosylation appears to promote ER localization of NFE2L3 as well, but this has been less explored (46). O-GlcNAcylation at Ser/Thr residues may also negatively regulate the transcriptional activity of NFE2L1, while positively regulating that of BACH2 (106, 107). Thus, sugar metabolism has a multifaceted impact on the activity of some CNC proteins. Of note, relatively few studies have explored the importance of specific CNC protein domains, protein-protein interactions, and PTMs in ferroptosis regulation.

Below, we merge the concepts introduced above to consider in detail the function of three CNC proteins for which there exists substantial published work in relation to ferroptosis: NFE2L2, BACH1, and NFE2L1 (Fig. 2*C*). The function of the remaining mammalian family members (NFE2L3, BACH2, and NFE2) in ferroptosis regulation remains largely or entirely unexplored; however, later in the review we note putative or known targets of these transcription factors that may regulate ferroptosis sensitivity.

## Regulation of ferroptosis sensitivity by NFE2L2

NFE2L2 is by far the most extensively studied CNC transcription factor both in general and in the context of ferroptosis (Fig. 2, *B* and *C*). Consistent with the understanding of NFE2L2 as an “antioxidant” transcription factor, it generally protects cells from ferroptosis. Cancer cell lines harboring mutations in *KEAP1*, a negative regulator of NFE2L2, are typically more resistant to small molecule inducers of ferroptosis than *KEAP1* wild-type cell lines (50, 108). *NFE2L2* knockdown or gene disruption also sensitizes lung adenocarcinoma cells, hepatoma cells, rat and human glioma cells, and ovarian cancer cells to ferroptosis (50, 109, 110, 111). *NFE2L2* overexpression confers ferroptosis resistance in many of these same cell types (29, 50, 112), though not all (110). In mice, Nfe2l2 also seems to protect from ferroptosis induced by intestinal ischemia/reperfusion (113), but the role of NFE2L2 in ferroptosis regulation beyond cultured cancer cells is generally less well understood.

NFE2L2 likely promotes ferroptosis resistance through several pathways (Fig. 3). One mechanism by which NFE2L2 can inhibit ferroptosis is through increased GSH synthesis (50). NFE2L2 positively regulates the expression of the GSH biosynthetic genes glutamate-cysteine ligase catalytic subunit (*GCLC*) and glutamate-cysteine ligase modifier subunit (*GCLM*) and increases GSH abundance in many cell types and contexts (50, 80, 82, 109, 111, 113, 114, 115, 116, 117, 118, 119). However, this function is not universal: in other cells, NFE2L2 does not appear to regulate GCLM (50) or GSH abundance (120). GSH is the cosubstrate of GPX4, a key antiferroptotic protein; thus, processes that increase GSH levels would tend to inhibit ferroptosis. Indeed, GCLC inhibition by buthionine sulfoximine can sensitize cells to ferroptosis (50, 121). NFE2L2 can also directly upregulate solute carrier family 7 member 11 (*SLC7A11*), the amino acid-transporting subunit of the system x_c_^-^ cystine/glutamate antiporter (note: system x_c_^-^ is the target of the ferroptosis-inducing molecule erastin) (110, 111, 113, 122, 123). Cystine import by SLC7A11 is important for the synthesis of GSH and possibly other sulfur-containing metabolites that inhibit ferroptosis (17, 124, 125). These data suggest NFE2L2 can confer ferroptosis resistance *via* increased cystine import and glutathione synthesis.

Despite the antiferroptotic effect of GSH, stimulation of the cystine/GSH/GPX4 axis alone does not seem to fully explain how NFE2L2 inhibits ferroptosis. For example, *Slc7a11* overexpression only partially reverts the sensitization to erastin observed in rat glioma cells in which *Nfe2l2* has been knocked down (110). In other cells, *SLC7A11* gene disruption sensitizes cells to ferroptosis, but the additional loss of *KEAP1* is sufficient to fully rescue this ferroptosis phenotype (17). This suggests that NFE2L2 hyperactivation, following *KEAP1* loss, can confer ferroptosis resistance through an SLC7A11-independent mechanism. All together, these results indicate that NFE2L2 gene targets besides those involved in cystine import are sufficient to inhibit ferroptosis.

NFE2L2 may also regulate ferroptosis sensitivity by decreasing the size of the intracellular labile iron pool (LIP). Generally, high levels of labile iron promote lipid peroxidation and ferroptosis (5, 126, 127). NFE2L2 reduces the size of the LIP in human cells and in mice under basal conditions and/or in the context of lipid peroxidation (109, 111, 116, 117, 118, 128, 129). NFE2L2 may influence the LIP by regulating the expression of the iron exporter solute carrier family 40 member 1 (*SLC40A1*, also known as ferroportin) (130). However, this mode of regulation appears context-dependent: while NFE2L2 upregulates *SLC40A1* expression in primary human macrophages treated with the GPX4 inhibitor RAS-selective lethal 3 (RSL3) (128), NFE2L2 downregulates *SLC40A1* expression in ovarian cancer cells—this could constitute a rare example of direct repression by NFE2L2 (130). Ferritin can sequester iron from the LIP. NFE2L2 can bind both ferritin heavy chain 1 (*FTH1*) and ferritin light chain (*FTL*) genes (80, 83). Moreover, NFE2L2 often upregulates *FTH1* mRNA and/or protein levels basally and/or in ferroptosis-inducing conditions (108, 109, 123, 129), which would tend to limit the size of the LIP. However, even in some cells where NFE2L2 upregulates *FTH1* mRNA levels, reduced FTH1 protein levels are observed (111), possibly due to the stimulation of ferritin turnover (see below). It is less clear whether or how NFE2L2 influences *FTL* expression, as NFE2L2 does not seem to regulate *FTL* mRNA levels in hepatoma cells (109) and negatively regulates *FTL* mRNA and protein levels in human cells and in mice (111, 116). Thus, while NFE2L2 appears to regulate ferritin through effects on FTH1, this regulation may be direct and/or indirect, context-dependent, and potentially balanced by distinct regulation of *FTL*.

Ferritin can be degraded through the process of ferritinophagy, resulting in the release of labile iron. As with iron export and storage, NFE2L2 seems to have a context-dependent role in ferritinophagy. Nuclear receptor coactivator 4 (NCOA4) is a key ferritinophagy cargo receptor (131). NFE2L2 directly binds the *NCOA4* gene in osteosarcoma cells (80). In RSL3-treated head and neck cancer (HNC) cells, NFE2L2 upregulates NCOA4 protein levels (129). However, *NCOA4* knockdown does not impact the LIP in these cells, despite sensitizing the cells to RSL3 (129). Thus, while NFE2L2 appears to positively regulate *NCOA4*, it is unclear whether *NCOA4* expression impacts the LIP in HNC cells. In contrast to HNC cells, NFE2L2 downregulates *NCOA4* mRNA and protein levels in ovarian cancer cells, both basally and in ferroptosis-inducing conditions (111). Moreover, NFE2L2 directly binds and promotes the expression of *HERC2*, which encodes an E3 Ub ligase that can target NCOA4 (111). These changes should inhibit ferritinophagy and decrease the LIP; however, NFE2L2 also indirectly upregulates the expression of *VAMP8*, which promotes ferritinophagy (111). While layered and complex, NFE2L2 ultimately decreases the LIP and ferroptosis sensitivity in ovarian cancer cells (111).

Additional connections exist between NFE2L2 and iron metabolism, and these are also complicated. Another canonical target of NFE2L2 is heme oxygenase 1 (*HMOX1*, this protein referred to as HMOX1 or HO-1) (80, 83, 84). NFE2L2 positively regulates *HMOX1* mRNA and protein levels in many contexts (109, 113, 116, 118, 129), though not all (50, 113, 129). HMOX1 breaks down heme into Fe^2+^, biliverdin, and carbon monoxide. Based on this function, HMOX1 might be expected to sensitize cells to ferroptosis by increasing the LIP. However, the answer is not so straightforward: *HMOX1* expression confers ferroptosis resistance in some cells (109) and ferroptosis sensitivity in others (132). Altogether, due in part to the complex role of HMOX1, a consistent role for NFE2L2-mediated *HMOX1* upregulation in ferroptosis regulation remains elusive.

Related to its role in iron metabolism, NFE2L2 may also regulate ferroptosis through upregulation of the metal binding protein metallothionein 1G (*MT1G*). In hepatoma cells, *MT1G* knockdown exacerbates lipid peroxidation upon treatment with the ferroptosis inducer sorafenib and further decreases GSH levels in erastin- or sorafenib-treated cells (114). Genetic or pharmacologic NFE2L2 inhibition largely abolishes sorafenib-induced increases in *MT1G* mRNA and protein levels (114, 133). These results suggest that upon ferroptosis induction, NFE2L2 directly and/or indirectly upregulates *MT1G*, which in turn confers ferroptosis resistance. However, the precise role of MT1G in ferroptosis regulation has yet to be elucidated.

NAD(P)H quinone dehydrogenase 1 (*NQO1*) is another canonical NFE2L2 target gene with potential roles in ferroptosis regulation (80, 83, 84). Ferroptosis inducers, including erastin, can increase NFE2L2 transcriptional activity as well as *NQO1* mRNA levels (109, 115). In hepatoma cells, erastin induces *NQO1* expression in an NFE2L2-dependent manner, and knocking down *NQO1* enhances erastin-induced growth arrest (109). This implies NFE2L2-mediated *NQO1* upregulation promotes ferroptosis resistance. However, in general, it remains somewhat unclear what role NQO1 might play in ferroptosis regulation, as it is uncertain which substrate(s) of NQO1 are relevant to the ferroptosis mechanism.

One question that warrants further study is whether NFE2L2 can modulate ferroptosis sensitivity through regulation of lipid synthesis. Pretreatment with the putative Nfe2l2 inhibitor ML385 increases Acsl4 protein levels in HT-22 murine neuronal cells treated with a ferroptosis inducer and an antioxidant (134). As ACSL4 increases ferroptosis sensitivity through its role in PUFA metabolism, these results are consistent with an antiferroptotic role for NFE2L2. However, Acsl4 levels were not analyzed upon ML385 treatment alone. Thus, whether Acsl4 is repressed by Nfe2l2 basally, and whether this regulation (if it occurs) is direct or indirect, remains to be determined. Nfe2l2 may also regulate the synthesis of antiferroptotic MUFAs. Young *Nfe2l2* KO mice express higher levels of hepatic *Scd* mRNA than control mice, though this phenotype was no longer observed in older mice (135). These results suggest Nfe2l2 may negatively regulate *Scd* expression, which would tend to decrease phospholipid MUFA abundance and increase ferroptosis sensitivity. However, the significance of this putative interaction in the context of ferroptosis has not been directly tested.

Finally, NFE2L2 may modulate ferroptosis sensitivity through direct regulation of genes encoding the two major enzymatic negative regulators of ferroptosis, GPX4 and FSP1. Nfe2l2 seems to directly upregulate *Gpx4* expression in rat kidney cells (136). NFE2L2 can also upregulate *GPX4* mRNA and/or protein levels in other cells (108, 112, 116, 137), though not all (108, 123). It is unknown whether NFE2L2 upregulates *GPX4* directly or indirectly in these cases. Notably, in HT-1080 fibrosarcoma cells and H1299 lung cancer cells, *NFE2L2* overexpression or *KEAP1* gene disruption can promote ferroptosis resistance even in cells lacking GPX4 (17, 50). This suggests NFE2L2 can protect cells from ferroptosis in a GPX4-independent manner. One such mechanism may involve FSP1. NFE2L2 can bind the *AIFM2/FSP1* promoter (17, 122) and upregulate *AIFM2/FSP1* expression (17, 108). FSP1 provides a clear example of ferroptosis regulation by the downstream target of a CNC family member: in lung cancer cells, NFE2L2 appears to promote ferroptosis resistance primarily by increasing FSP1 abundance. Here, *KEAP1* loss increases FSP1 levels and decreases ferroptosis sensitivity, presumably through NFE2L2 stabilization (17). These ferroptosis phenotypes are abolished upon genetic or pharmacologic FSP1 inhibition (17). Moreover, *AIFM2/FSP1* overexpression is sufficient to rescue ferroptosis resistance in cells lacking NFE2L2 (17). Thus, NFE2L2 may inhibit ferroptosis in lung cancer cells mainly through increased *AIFM2/FSP1* expression.

Despite the evidence that NFE2L2 potently suppresses ferroptosis in many contexts, this finding is not universal. For example, in *KEAP1* gene-disrupted HAP1 haploid cells, NFE2L2 stabilization is not associated with substantial ferroptosis resistance (138). Likewise, in T98G glioblastoma cells, which exhibit high basal *NFE2L2* expression, NFE2L2 appears to increase ferroptosis sensitivity (120). These phenotypes seem to be explained by increased expression of ATP binding cassette subfamily C member 1 (*ABCC1*) (encoding the multidrug resistance-associated protein 1, MRP1), a canonical NFE2L2 target gene (120, 139). Mechanistically, MRP1 exports glutathione and glutathione conjugates from the cell, decreasing intracellular levels of this protective metabolite and thereby increasing ferroptosis sensitivity (138). Thus, stabilized NFE2L2 in *KEAP1*-mutant cells may exert both proferroptotic and antiferroptotic effects (138). Finally, in rat glioma cells, *Nfe2l2* overexpression confers resistance to RSL3, but not erastin (110). How the antiferroptotic and proferroptotic mechanisms downstream of NFE2L2 are balanced, and why the balance differs among cell lines and conditions, remains to be fully explored.

An important line of inquiry centers around how ferroptosis induction itself alters NFE2L2 levels and function. That is, might there exist a feedback loop in which NFE2L2 negatively regulates ferroptosis, and ferroptosis induction in turn regulates NFE2L2 stabilization? Indeed, in many cases, ferroptosis-inducing stimuli promote NFE2L2 stabilization, presumably resulting in greater ferroptosis resistance through the pathways described above. In primary human macrophages, RSL3 treatment increases NFE2L2 abundance (128). In hepatoma cells, treatment with erastin or sorafenib increases NFE2L2 protein levels in both nuclear and whole cell extracts, as well as NFE2L2 transcriptional activity and NFE2L2 cofactor binding (109). Mechanistically, these ferroptosis inducers enhance NFE2L2 levels by decreasing KEAP1-NFE2L2 protein interactions and downregulating KEAP1 levels (109). Consistent with these observations, cisplatin-resistant HNC cells treated with GPX4 inhibitors exhibit decreased KEAP1 protein levels and increased NFE2L2 protein levels resulting from a posttranscriptional mechanism (129). NFE2L2 stabilization upon ferroptosis induction likely constitutes a mechanism by which cells respond to and protect themselves from ferroptosis. However, in at least one example, increased membrane lipid peroxidation may promote NFE2L2 cytosolic sequestration and thereby NFE2L2 inactivation as a result of association with caveolae associated protein 1 (CAVIN1) (140). Defining positive and negative feedback loops connecting NFE2L2 and ferroptosis will help to understand how ferroptosis sensitivity is tuned after the onset of lipid peroxidation.

## Regulation of ferroptosis sensitivity by BACH1

In contrast to NFE2L2, BACH1 functions primarily as a transcriptional repressor (141, 142). Indeed, in many contexts, NFE2L2 and BACH1 exert opposite effects on the expression of common target genes (128). Accordingly, BACH1 enhances ferroptosis sensitivity in many cell types, including mouse embryonic fibroblasts (MEFs), glioma cells, esophageal squamous cell carcinoma (ESCC) cells, and primary human macrophages (128, 143, 144, 145). The proposed mechanisms by which BACH1 sensitizes cells to ferroptosis are highly context-dependent, varying by cell type and condition; indeed, some evidence suggests BACH1 can confer ferroptosis resistance (146). However, existing data generally support a model in which BACH1 increases ferroptosis sensitivity through transcriptional regulation of glutathione metabolism, iron transport and storage, and fatty acid metabolism (Fig. 3).

Chromatin immunoprecipitation studies indicate that BACH1 can bind *GCLC*, *GCLM*, *GPX4*, and *SLC7A11*—all known ferroptosis regulators (147, 148, 149, 150). However, target gene binding appears to be context- or cell type-specific, as Bach1 does not seem to bind *Gpx4* in murine M1 cells (143). BACH1 negatively regulates *GCLM*, *SLC7A11*, and *GPX4* expression as well as GSH abundance in diverse cell types and conditions (143, 147, 148, 150, 151, 152, 153), albeit with the same caveat that this does not always appear to be the case (144, 148, 150). In a mouse model of cerebral artery occlusion, *Bach1* expression decreases protein levels of Gpx4 and Slc7a11, as well as GSH abundance (152). Interestingly, this repressive mechanism is indirect and is proposed to involve a KDM4C-COX2 signaling axis that remains somewhat undefined (152). As is true for NFE2L2, regulation of the cystine/GSH/GPX4 axis does not seem to fully explain how BACH1 modulates ferroptosis sensitivity. While *Bach1* gene-disrupted MEFs exhibit higher expression of *Gclc*, *Gclm*, and *Slc7a11*, knocking down any one of these genes individually does not restore ferroptosis sensitivity (143). It may be that repression of multiple genes is required for BACH1 to mediate ferroptosis sensitization. Alternatively, regulation of non GSH-related genes may be necessary for ferroptosis sensitization by BACH1.

Like NFE2L2, BACH1 may also regulate iron metabolism, either basally or in response to proferroptotic stimuli. BACH1 can bind and negatively regulate the expression of iron transport- and storage-related genes (*e.g.*, *FTH1*, *FTL*, *SLC40A1*, and *HMOX1*) in multiple cell types and conditions (128, 143, 146, 147, 148, 149, 150, 151, 154, 155). However, Bach1 does not appear to regulate *Ftl* basally in immortalized MEFs (148). In lipid peroxidation-inducing conditions, BACH1 positively regulates intracellular and mitochondrial iron abundance (147, 148, 151, 152), though Bach1 may repress basal mitochondrial iron levels in MEFs (143). In rat neural progenitor cells, *Bach1* knockdown partially rescued phenotypes induced by the oxidant tert-butyl hydroperoxide, including increases in lipid peroxidation, Fe^2+^, and *Acsl4* mRNA and protein levels, and decreases in GSH, *Gpx4*, *Slc7a11*, *Fth1*, *Ftl*, and *Hmox1* mRNA and protein abundance (147). In these *Bach1* knockdown cells, *Hmox1* gene silencing partially restored *Bach1* knockdown-induced effects on lipid peroxidation and GSH, whereas *Gpx4* knockdown partially restored the effects on Fe^2+^, Ftl, Fth1, Slc7a11, and Acsl4 levels (147). This is consistent with the notion that no single pathway alone (*e.g.*, GSH or iron metabolism) is sufficient for ferroptosis sensitization by BACH1. Instead, BACH1 may regulate multiple pathways in parallel to exert its proferroptotic function.

BACH1 may also regulate ferroptosis through effects on lipid metabolism. As noted above, PUFAs promote ferroptosis, while MUFAs suppress ferroptosis. In ESCC cells, *BACH1* overexpression increases the abundance of PUFA-containing phospholipids and decreases the levels of MUFA-containing phospholipids, thereby increasing sensitivity to GPX4 inhibition (144). Supplementation with the MUFA oleic acid largely restores RSL3 resistance in these cells (144). Indeed, BACH1 can bind and negatively regulate the expression of *SCD* (144). Consistent with its known function, *SCD* knockdown increases lipid peroxide abundance in RSL3-treated ESCC cells (144). Thus, BACH1 may sensitize ESCC cells to ferroptosis by repressing *SCD* and thereby inhibiting MUFA synthesis.

BACH1 may also regulate other genes to promote ferroptosis sensitivity in certain cell types. For instance, in human pancreatic cancer cells, BACH1 binds the *AIFM2/FSP1* gene, though Bach1 does not bind *Aifm2/Fsp1* or regulate its expression in murine cells (148). In another example, BACH1 levels can correlate positively with expression of *ACSL4* and the NADPH oxidase *NOX1* (154), which together would favor the generation of lipid peroxides. The nature of these relationships, including whether *ACSL4* and *NOX1* are direct BACH1 targets, has not yet been elucidated.

Ferroptosis induction generally coincides with decreased BACH1 protein levels. In primary human macrophages, RSL3 treatment decreases the abundance of nuclear BACH1 (128). In MEFs, erastin treatment increases *Bach1* mRNA levels but seems to promote Bach1 protein degradation through proteasomal and nonproteasomal mechanisms (143). Oxidants that are thought to be inducers of ferroptosis, cumene hydroperoxide and tert-butyl hydroperoxide, can also promote the proteasome-dependent degradation of BACH1 (146). Similarly, BACH1 degradation is increased by isoproterenol, another compound that may be able to directly induce ferroptosis (155). The mechanism by which ferroptosis promotes BACH1 nuclear export and degradation has yet to be defined. Regardless, the reduction in BACH1 abundance should decrease ferroptosis sensitivity. One hypothesis is that this negative feedback between ferroptosis induction and BACH1 expression is part of a homeostatic mechanism to suppress cell death.

## Regulation of ferroptosis sensitivity by NFE2L1

Most experimental attention has focused on NFE2L2 (Fig. 2, *B* and *C*). However, *Nfe2l1* is the only CNC family member that is essential for mouse embryonic development (156). By contrast, *Nfe2l2* is dispensable for this process, albeit required for defense against stresses including drug-induced glutathione depletion (157, 158, 159, 160, 161). These data suggest nonredundant roles for CNC family members despite reported overlap in target genes. Indeed, while NFE2L2 is known for its role in mitigating oxidative stress, NFE2L1 is best known for maintaining proteostasis (50, 55, 162). However, NFE2L1 can also upregulate the expression of GSH biosynthetic genes (*GCLC*, *GCLM*, and glutathione synthetase [*GSS*]) and thereby regulate cellular antioxidant responses (92, 163, 164, 165). That said, new results suggest that NFE2L1 can regulate ferroptosis through mechanisms that are distinct from those related to GSH synthesis.

To date, evidence that NFE2L1 can regulate ferroptosis sensitivity comes from a limited number of models, including cultured human A549 non–small cell lung cancer cells and murine adipose tissue. In A549 cells, genetic disruption of *NFE2L1* or its upstream positive regulator *NGLY1* results in heightened ferroptosis sensitivity (50). Notably, NFE2L1 loss sensitizes these cells to ferroptosis despite high constitutive NFE2L2 levels resulting from a *KEAP1* mutation. GPX4 is necessary for NFE2L1-mediated ferroptosis resistance in HT-1080 fibrosarcoma cells and sufficient to rescue ferroptosis resistance in *NFE2L1* gene-disrupted A549 cells (50) (Fig. 4). However, NFE2L1 does not seem to upregulate *GPX4* mRNA levels in A549 cells (50). This may not be universal as NFE2L1 can bind the *GPX4* promoter in osteosarcoma cells (80). These data highlight an important role for GPX4 in NFE2L1-mediated ferroptosis resistance in human cancer cells but with the specific mechanistic connection between these regulators still poorly defined.

Further insights into NFE2L1-dependent ferroptosis regulation have been obtained from murine cells and animal models. Consistent with results from A549 cells, *Nfe2l1* knockdown in immortalized murine brown adipocytes does not alter *Gpx4* mRNA levels (166). Unlike A549 cells, *Nfe2l1*-deficient murine brown adipose tissue (BAT) generally contains higher Gpx4 protein levels compared to control BAT (166). Notably, however, Gpx4 protein is more highly ubiquitinated in *Nfe2l1*-deficient BAT than in control tissue (166). One model to reconcile the A549 and murine BAT data is that ubiquitinated Gpx4 accumulates upon Nfe2l1 loss in murine brown adipocytes but not in A549 cells (*e.g.*, adipocytes have a defect in protein degradation while A549 cells do not). Putative ubiquitination sites that promote GPX4 proteasomal degradation have been identified, though the mechanisms targeting GPX4 for ubiquitin-mediated proteolysis have yet to be fully elucidated (167). It is also possible that proteins other than Gpx4 mediate ferroptosis regulation by Nfe2l1. In addition to Gpx4, *Nfe2l1*-deficient BAT exhibits higher levels and ubiquitination of other antiferroptotic proteins, including Gclm, Gclc, and Gss (166). The significance of these phenotypes in the context of NFE2L1-mediated ferroptosis regulation is unknown.

NFE2L1 may regulate the abundance of downstream ferroptosis regulators indirectly. For example, in human oral squamous cell carcinoma cells, NFE2L1 can increase ferroptosis resistance through the Holliday junction recognition protein (HJURP) (168) (Fig. 4). NFE2L1 binds the *HJURP* promoter and increases its expression, as well as protein levels of SLC7A11 and GPX4 (168). The NFE2L1-mediated increase in GPX4 and SLC7A11 protein appears to be at least partially HJURP-dependent, demonstrating potential indirect mechanisms by which NFE2L1 regulates ferroptosis (168). However, further study is needed to clarify the role HJURP plays in this process.

An interesting question centers around how NFE2L1 itself might be regulated under ferroptosis-inducing conditions. In immortalized murine brown adipocytes, Nfe2l1 levels increase following treatment with the GPX4 inhibitors RSL3 or FIN56, but not the system x_c_^-^ inhibitor erastin (166). The accumulation of NFE2L1 protein in response to ferroptosis-inducing molecules is context-specific; in A549 cells, neither the GPX4 inhibitor ML162 nor the system x_c_^-^ inhibitor erastin2 increase NFE2L1 abundance (50). There is also no known mechanism linking GPX4 inhibition to increased NFE2L1 protein abundance and function. However, one intriguing model could be that ER membrane lipid peroxidation, an early event in the ferroptosis mechanism (12, 26, 169), directly impacts the synthesis or processing of NFE2L1 at this organelle.

There are many open questions regarding ferroptosis regulation by NFE2L1. First, in contrast with A549 cells, *NGLY1* gene disruption in HepG2 hepatoma cells and MEFs causes no change or even an increase in resistance to erastin2 (50). The explanation for these context-specific differences is unknown. Second, chromatin immunoprecipitation assays demonstrate that NFE2L1 binds the genes *GCLC*, *FTH1*, *ABCC1*, *FTL*, *HMOX1*, and *AIFM2/FSP1* in osteosarcoma cells (80). However, NFE2L1 does not appear to regulate GCLC or GCLM protein levels in A549 cells (50). Further work is needed to determine whether NFE2L1 can, in some contexts, modulate ferroptosis sensitivity through regulation of these genes. Third, multiple *NFE2L1* mRNA isoforms exist, and the function of each isoform in ferroptosis regulation has not been examined. Human cells express several isoforms, including a longer isoform (termed *TCF11*) and a shorter isoform (*Nrf1α*), which lacks the Neh4 domain (170, 171) (Fig. 2*A*). Murine cells also express isoforms including *Nrf1α*, but unlike human cells, they do not express *TCF11* (171). To fully understand how NFE2L1 regulates ferroptosis sensitivity it will likely be necessary to define the function of each NFE2L1 isoform in different cells and contexts.

## Interplay between CNC transcription factors in ferroptosis regulation

Above, we have mostly described the functions of CNC transcription factors in isolation. However, these proteins may also interact with one another to regulate ferroptosis. As mentioned above, NFE2L2 and BACH1 generally exert opposing effects on ferroptosis sensitivity. These two transcription factors may, in fact, be in direct competition for the regulation of common target genes. It is also possible that BACH1 regulates NFE2L2 abundance. Lung epithelial cells treated with lipopolysaccharide (LPS) exhibit cell death that resembles ferroptosis. In LPS-treated cells, knocking down *BACH1* decreases the levels of iron, ROS, lipid peroxidation, and cell death (151). In these cells and others, BACH1 loss results in increased NFE2L2 protein levels (151, 154). (It should be noted that neither study measured *NFE2L2* mRNA levels.) Consistent with a model in which BACH1 loss increases NFE2L2 levels and activity, pharmacologic NFE2L2 inhibition reverts *BACH1* knockdown-induced decreases in ROS and iron levels in LPS-treated cells (151). NFE2L2 inhibition also reverts *BACH1* knockdown-induced increases in GPX4 and SLC7A11 protein levels (151). Thus, BACH1 may regulate the abundance of antiferroptotic proteins and lipid peroxidation by suppressing NFE2L2 through a mechanism that has yet to be determined.

Additional evidence suggests ferroptosis sensitivity may be regulated by linked chains of regulation involving multiple CNC proteins. In osteosarcoma cells, NFE2L1 can bind the third intron of *NFE2L3* and NFE2L3 can bind the first intron and promoter of *NFE2L2* (80). Thus, NFE2L1 may be able to directly regulate the expression of *NFE2L3*, and NFE2L3 may in turn be able to regulate *NFE2L2* expression. CNC proteins may also indirectly regulate the levels of other CNC proteins. For example, NFE2L3 can indirectly repress NFE2L1 translation (172). In another example, NFE2L2 can stabilize BACH1 indirectly through *HMOX1* upregulation in lung cancer cells, likely by reducing heme levels (173). As such, ferroptosis-related phenotypes that appear to be driven by a single CNC transcription factor may reflect complex interactions between CNC members. A deeper understanding of all CNC members and their interrelationships will help clarify the role CNC proteins play in ferroptosis regulation.

An interesting and seemingly complex example of interactions between CNC proteins relates to NFE2L2 and NFE2L1. A549 cells have high basal levels of NFE2L2 due to a mutation in *KEAP1*. Disrupting *NFE2L2* in these cells results in substantial ferroptosis sensitization, showing that NFE2L2 confers ferroptosis resistance in these cells (50). However, despite high NFE2L2 protein levels in these *KEAP1*-mutant cells, *NFE2L1* disruption is sufficient to sensitize these cells to ferroptosis (50). This implies that NFE2L2 and NFE2L1 have unique targets that are independently important for ferroptosis regulation. Indeed, disruption of *NFE2L2*, but not *NFE2L1*, depletes GSH (50). However, *NFE2L2* overexpression in *NFE2L1* gene-disrupted A549 cells nearly restores ferroptosis resistance to the level of control cells (50). How *NFE2L2* overexpression can compensate for NFE2L1 loss is not yet clear. It is possible that supranormal expression of NFE2L2-regulated target genes (*e.g.*, *GCLC* and *GCLM*) is sufficient to compensate for the loss of NFE2L1. Alternatively, it could be that when the abundance of NFE2L2 surpasses some threshold, it can regulate the expression of additional genes for which it has weaker affinity (*e.g.*, a gene normally regulated only by NFE2L1).

On the other hand, NFE2L1 cannot fully compensate for the loss of NFE2L2 in A549 cells. *NFE2L1* overexpression in *NFE2L2*-disrupted cells only partially rescues ferroptosis resistance (50). This could be because NFE2L1 is more restricted in its ability to regulate canonical NFE2L2 target genes in A549 cells. Or perhaps, even though NFE2L1 protein levels are increased upon *NFE2L1* overexpression, much of the NFE2L1 protein may be retained in the ER and therefore unable to translocate to the nucleus at levels greater than control cells.

The functions of NFE2L1 and NFE2L3 may also be interdependent. NFE2L3 shares a highly similar domain architecture with NFE2L1 and, like NFE2L1, may be regulated by NGLY1-dependent deglycosylation (Fig. 2*A*). The tissue expression pattern of *NFE2L3* is relatively restricted compared to *NFE2L1*, with particularly high expression in the placenta (174). That said, *NFE2L3* is often highly expressed in cancer cells compared to normal tissue (175, 176). The structural similarities between NFE2L1 and NFE2L3 raise the possibility that NFE2L1 and NFE2L3 could function redundantly in some contexts—a model supported by some data (172). This redundancy could theoretically extend to ferroptosis regulation, though this concept has not yet been explored.

CNC members can also interact with transcription factors outside the CNC family to regulate ferroptosis sensitivity. BACH1 physically interacts with p53^R175H^, a p53 mutant protein expressed in some cancer cells (150). p53^R175H^ inhibits BACH1-mediated *SLC7A11* repression in cancer cells (150). Consistent with this, in cells harboring *p53*^*R175H*^, CRISPR/Cas9-mediated p53 loss sensitizes to ferroptosis through a BACH1-dependent mechanism (150). Mechanistically, BACH1 forms a protein complex with p53^R175H^ and the lysine-specific histone demethylase 1B (LSD2) at the *SLC7A11* promoter, leading to *SLC7A11* upregulation (150). These findings illustrate how CNC and non-CNC transcription factors can interact to regulate ferroptosis and may prove to be only one of many similar examples.

## Conclusions and future directions

CNC-family proteins can regulate ferroptosis through several pathways, including those involved in glutathione biosynthesis and metabolism, iron storage and metabolism, and lipid metabolism. Critically, much of the existing data remains correlational (*e.g.*, perturbation of CNC-family gene/protein expression, yielding some downstream changes in gene expression and ferroptosis sensitivity). Our understanding would improve with more insights into which specific target gene, or combination of target genes, is necessary and sufficient to modulate ferroptosis sensitivity downstream of each CNC protein. However, as noted throughout this review, transcriptional regulation by CNC proteins often seems to differ depending on the cell type or context. For this reason, it is difficult to predict CNC protein function in one cell type based only on results from another cell type. Published data could be used to formulate a particular hypothesis related to GSH, iron, or lipid metabolism, but the prevalence of context-specific regulation seemingly indicates that experimental studies will always be required in new model systems.

Most experimental attention to date has focused on NFE2L2, BACH1, and NFE2L1. However, emerging studies point to roles for the other CNC members in ferroptosis regulation. For example, BACH2 may increase ferroptosis sensitivity in immortalized human kidney cells through direct repression of *SLC7A11* and *GPX4* (106). *BACH2* knockdown partially rescues changes in iron and GSH levels induced by erastin treatment, suggesting BACH2 may regulate ferroptosis through both glutathione- and iron-related mechanisms (106). Some target genes of NFE2 and SKN-1 (the CNC family member expressed in *C. elegans*) also have known roles in ferroptosis regulation, such as those involved in GSH metabolism (149, 177). NFE2L3 also regulates ferroptosis-related genes, including those involved in glutathione biosynthesis (80). We speculate that these CNC transcription factors likely also regulate ferroptosis sensitivity, but further investigation is needed.

## Conflict of interest

S. J. D. is an inventor on patents related to ferroptosis. The other author declares that they have no conflicts of interest with the contents of this article.
